# COVID-19 vaccine or booster uptake and hesitancy for children aged 6 months–5 years in the United States: A national descriptive study using the household pulse survey between March and May 2023

**DOI:** 10.1016/j.jvacx.2024.100582

**Published:** 2024-11-07

**Authors:** Chulwoo Park, Pyramida Vagoyan Zabala

**Affiliations:** Department of Public Health and Recreation, San José State University, San Jose, CA 95192, United States

**Keywords:** COVID-19 vaccine, Updated COVID-19 booster, Vaccine hesitancy, Children aged 6 months–5 years (children under 5), Descriptive study, Test for proportions

## Abstract

The Centers for Disease Control and Prevention recommends that children aged 6 months–5 years (children under 5) receive multiple doses of COVID-19 vaccine, including updated (bivalent) COVID-19 vaccine. However, since the updated vaccines became available for children under 5, there has been limited knowledge regarding hesitancy toward the original vaccine or boosters in vaccinating children. To address this gap, this study utilized data from the Household Pulse Survey conducted from March 1, 2023 to May 8, 2023 (Phase 3.8). The purpose of the study was to examine the proportion of children under 5 who had received at least one dose of COVID-19 vaccine and to assess the likelihood of vaccination, categorized as willingness to vaccinate, moderate hesitancy, and strong hesitancy. Additionally, we identified sociodemographic factors that may influence the level of vaccine hesitancy for children. Overall, 24.5 % (95 % CI: 22.2–26.8) of children under 5 had received at least one dose of the COVID-19 vaccine, with the highest percentage among non-Hispanic Asians (42.1 %, 95 % CI: 36.4–47.8). Respondents who had not received the vaccine showed a statistically significant decrease in their inclination to vaccinate children. There was a higher level of vaccine hesitancy among females compared to males regarding vaccinating children under 5. As household income and educational attainment decreased, there was an observed increase in the proportion of individuals showing strong vaccine hesitancy. Households without health insurance had significantly lower vaccine uptake and higher rates of strong vaccine hesitancy compared to those with health insurance. Parents, guardians, and healthcare providers play essential roles in vaccinating children under 5. Targeted interventions are needed to address vaccine hesitancy and promote the importance of booster doses in the vaccination of children.

## Highlights

• COVID-19 vaccine or booster hesitancy among children under 5 in the United States.
• Analyzed Phase 3.8 (Weeks 55–57) of the Household Pulse Survey data.
• Sociodemographic factors affect vaccination uptake and hesitancy patterns.
• Reasons for strong hesitancy: unnecessity, unwillingness, mistrust, and side effects.
• Targeted interventions needed for vaccine hesitancy among children under 5.

## Introduction

Vaccine hesitancy is defined as “delay in acceptance or refusal of vaccination despite availability of vaccination services,” which is a multifaceted phenomenon shaped by unique circumstances, varying over time, geographical locations, vaccine type, and perceptions of necessity and trust [1], [2], [3]. Willingness to get vaccinated also varies by racial/ethnic, political, and religious affiliation [4]. Although vaccination is recognized as a highly effective public health measure, an increasing number of people perceive it as unsafe and unnecessary [5]. This declining confidence in vaccines poses a significant challenge to vaccination programs, leading to reduced vaccine coverage and a heightened risk of outbreaks and epidemics of vaccine-preventable diseases [5]. Furthermore, parental perceptions and approaches to vaccination critically influence decisions about their children’s immunization. This influence is evident in the hesitancy surrounding the Human Papillomavirus vaccine, where parental concerns directly affect their children’s willingness to receive it [6], [7], [8]. Parental beliefs can significantly shape the attitudes and intentions of the younger generation, underscoring the essential role of parental perspectives in shaping vaccination behaviors and outcomes.

The Centers for Disease Control and Prevention (CDC) recommends everyone ages 6 months and older receive a COVID-19 vaccine to helps protect against disease, hospitalization, and death [9]. With the emergence of new COVID-19 variants and the authorization of updated vaccines, it is crucial to understand the various factors influencing the vaccination decision-making process for children. The original (monovalent) COVID-19 vaccines were first authorized under emergency use authorization by the U.S. Food and Drug Administration (FDA) in December 2020 to combat the rapid transmission of the virus across populations [10]. These original COVID-19 vaccines became available to children aged 6 months to 5 years (hereafter, children under 5) in June 2020 [10]. In April 2023, FDA replaced them with the COVID-19 updated (bivalent) vaccines, authorized under emergency use [11]. The updated vaccine became available for individuals aged 12 years and older on September 2, 2022, and for those aged 5–11 years on October 12, 2022 [12]. Moderna COVID-19 vaccine (bivalent, original and omicron BA.1) and Pfizer-BioNTech COVID-19 vaccine (bivalent, original and omicron BA.4/BA.5) protects individuals 6 months of age and older against COVID-19 and simplifies the timeline for vaccination [11], [12], [13]. Unvaccinated children under 5 may receive either a two-dose series of the Moderna bivalent vaccine or a three-dose series of the Pfizer-BioNTech bivalent vaccine [12]. Children under 5 previously vaccinated with the original (monovalent) vaccine may receive an updated vaccine (booster) where the number of doses would depend on the child’s age and vaccination history [11], [12].

As of May 2023, approximately 56 million children under 5 have received the COVID-19 bivalent booster vaccine, accounting for 18 % of this age group in the United States [14]. Concerns regarding the safety of COVID-19 vaccines have been a topic of ongoing controversy and debate throughout the pandemic, contributing to widespread vaccine hesitancy among parents, many of whom express distrust about vaccinating their children. This hesitancy is complex and influenced by multiple factors, including misinformation, political ideology, fears of potential side effects, concerns about long-term risk, and skepticism surrounding the expedited vaccine development process [15], [16], [17], [18], [19], [20], [21], [22], [23]. Parents of children under 5 in the United States have voiced concerns about vaccine safety, expressed distrust towards the government, science, or the pharmaceutical industry, questioned the necessity of the vaccine, or believed their children were too young for vaccination [19], [20], [24], [25], [26], [27].

The Theory of Planned Behavior, identification of three types of beliefs—behavioral belief, normative beliefs, and control beliefs—that shape an individual’s intention to engage in a specific behavior, could be applied to influence of parents’ and caregivers’ vaccination decisions for children under 5 [28]. Behavioral beliefs, such as perceived risks or benefits of the COVID-19 vaccine, can shape attitudes toward vaccinating young children. Normative beliefs, or perceptions of social pressures from family, healthcare providers, and peer groups, can impact the decision-making process by influencing individuals’ desire to conform to what they perceive as socially acceptable. Finally, control beliefs, or perceived ease or difficulty in accessing the vaccine, can play a critical role, especially for households with limited healthcare access or insurance coverage.

Despite the pressing need for information, a significant knowledge gap exists regarding the percentage of children under 5 in the United States who have received at least one dose of the COVID-19 vaccine, along with the factors influencing their likelihood of vaccination and the sociodemographic elements related to vaccine hesitancy. Using the most recent data from Phase 3.8 of the Household Pulse Survey (HPS) conducted by the U.S. Census Bureau (as of May 8, 2023), this study aimed to examine the current landscape of vaccine uptake and hesitancy among children under 5 in the United States. By analyzing sociodemographic information of households, the research sought to provide insights into the present situation.

## Material and methods

### Study design and data sources

The U.S. Census Bureau, in collaboration with other agencies, initiated HPS to regularly gather information on households’ experiences and impact of the COVID-19 pandemic across the country. The HPS data collection began between April 23, 2020 and July 21, 2020 (Phase 1) [29]. This ongoing survey uses the U.S. Census Bureau’s Master Address File (MAF) to reach a representative sample of households in the United States [30]. Utilizing this publicly available secondary data, we conducted a descriptive analysis of Phase 3.8 HPS data, collected from March 1, 2023, to May 8, 2023. To maintain consistency with Phase 1, Phase 3.8 retained the use of “Weeks” as the designated collection periods (Week 55: March 1–March 13, 2023, Week 56: March 29–April 10, 2023, and Week 57: April 26–May 8, 2023). We followed to the Strengthening the Reporting of Observational Studies in Epidemiology (STROBE) Statement guidelines for reporting observational studies [31].

### Analytic sample

**Fig. 1:**
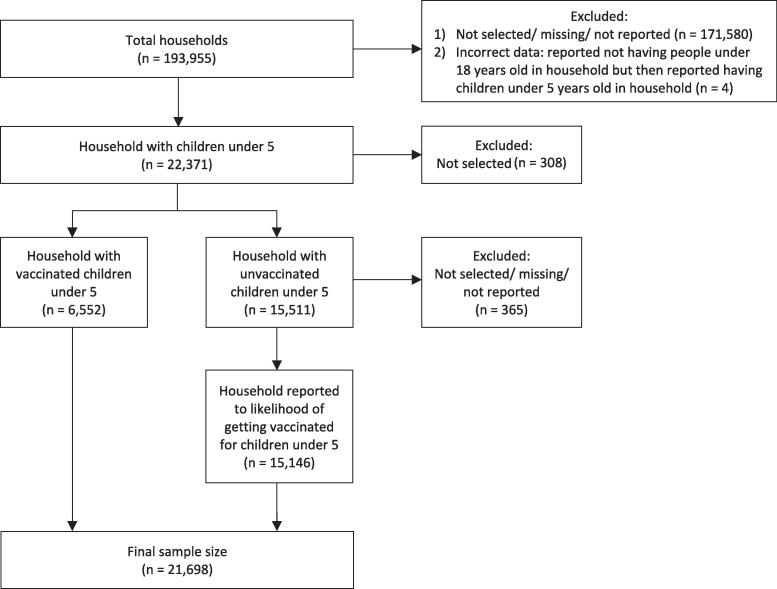
Analytic sample from Household Pulse Survey, Phase 3.8 (March 1, 2023–May 8, 2023).

Phase 3.8 of HPS utilized the July 2022 MAF for data collection [30]. The U.S. Census Bureau contacted sampled households via email and/or SMS during weekdays to administer HPS through the online data collection platform Qualtrics, and sent reminders to nonrespondents [30]. The survey informed respondents that their participation would help measure the impact of social and economic factors, including ongoing effects of COVID-19, to aid federal, state, and local agencies in identifying emerging community issues [32]. Federal law protected respondents’ privacy, ensuring their answers were kept confidential and not publicly disclosed in an identifiable way. Participation was voluntary, with no gift or monetary compensation offered, and the survey took approximately 20 min to complete [32]. When the household or individual had answered enough of the questionnaire, the interview was considered complete. Insufficient partial interviews were classified as nonrespondents [30]. For our analysis, we focused specifically on households with children under 5, drawing Phase 3.8 of HPS. The total number of respondents across the three collection periods was 193,955 (Week 55: 72,738, Week 56: 61,927, and Week 57: 59,290), with response rates of 6.7 %, 5.7 %, and 5.5 % respectively [30]. From this population, we selected households with children under 5 (n = 22,371) and further divided them into two groups: households with vaccinated children under 5 (n = 6,552) and households with unvaccinated children under 5 (n = 15,511), resulting in a final sample size of 21,698 (Fig. 1).

### Vaccine hesitancy measurement: questions for vaccine or booster hesitancy

Adult respondents aged 18 years and older from households with children under 5 were asked, “For the children in this household, how long ago was their most recent dose of the COVID-19 vaccine or booster?”. Response options included: “on or after December 9, 2022,” “before December 9, 2022 but less than a year ago,” “more than a year ago,” and “not vaccinated.” Respondents who reported that their children under 5 were not vaccinated were then asked to indicate the likelihood of vaccinating their children, with response options on a Likert scale: “definitely get the children a vaccine” (hereafter, definitely), “probably get the children a vaccine” (hereafter, probably), “be unsure about getting the children a vaccine” (hereafter, unsure), “probably NOT get the children a vaccine” (hereafter, probably NOT), “definitely NOT get the children a vaccine” (hereafter, definitely NOT), and “I do not know the plans for vaccination of the children under 5 in my household” (hereafter, don’t know). Respondents who selected probably, unsure, probably NOT, or definitely NOT were then asked to indicate reasons why the parents or guardians of children under 5 in their household had not yet vaccinated them. 10 possible reasons were provided, and respondents could select all that applied. We further categorized the likelihood of vaccination into three groups: 1) willingness to vaccinate (definitely or probably), 2) moderate hesitancy (unsure or probably NOT), and 3) strong hesitancy (definitely NOT).

In addition, the survey asked respondents about the timing of their most recent dose of the COVID-19 vaccine or booster, with response options: “on or after September 1, 2022,” “before September 1, 2022 but less than a year ago,” and “more than a year ago.” Respondents who selected “before September 1, 2022 but less than a year ago,” or “more than a year ago” were classified as having not received an updated COVID-19 vaccine booster dose and were asked to indicate reasons for this decision. Nine possible reasons were provided, allowing respondents to select all that applied.

### Quantitative variables

For sociodemographic information of households, we included the following categorical variables (binary, nominal, or ordinal):

• COVID-19 vaccination status: received COVID-19 vaccine (binary: yes, no)
• COVID-19 status: tested positive or informed by doctor or provider of having COVID-19 (binary: yes, no)
• Presence of current COVID-19 symptoms (binary: yes, no)
• Race/ethnicity: combined two original variables, Hispanic Origin and Race, into a single variable (nominal: non-Hispanic White, non-Hispanic Black, non-Hispanic Asian, non-Hispanic other race, and Hispanic)
• Sex at birth (binary: male, female)
• Age: categorized the original variable, Year of Birth, into distinct age groups (ordinal: aged 18–29, 30–39, 40–49, 50–59, 60–69, and 70 and over),
• Region (nominal: Northeast, South, Midwest, and West)
• Marital status (nominal: now married, widowed, divorced, separated, and never married)
• Educational attainment: combined categories of less than high school and some high school (ordinal: less than or some high school, high school graduate or equivalent, some college but no degree or is in progress, associate’s degree, bachelor’s degree, and graduate degree)
• Income (ordinal: less than $25,000, $25,000–$34,999, $35,000–$49,999, $50,000–$74,999, $75,000–$99,999, $100,000–$149,999, $150,000–$199,999, and $200,000 and above)
• Health insurance: merged two original variables, Private and Public, into a single variable indicating the presence or absence of any type of health insurance (binary: yes, no).

### Statistical methods

In conducting a descriptive analysis, we calculated proportions along with survey-weighted 95 % confidence intervals (CIs) using 80 replicate weights derived from successive difference replications. All figures (including Supplementary Material) and table presented in this study were based on survey-weighted proportions. When comparing each category with a selected reference group within a variable, we employed tests for proportions with a two-sided *P*-value threshold of <0.05, <0.01, and <0.001. Missing data were excluded on a variable basis, with the numbers of missing data ranging from 0 to 4,556. For quantitative analysis, we utilized Stata/MP 14.2. (StataCorp, College Station, TX).

## Results

### COVID-19 vaccine or booster uptake and intention to vaccination for children under 5

We examined the survey-weighted proportions of COVID-19 vaccine or booster uptake and the likelihood of vaccination or booster for children under 5, taking into account the sociodemographic factors of households (Table 1). When conducting tests for proportions, we used the first option within binary variables (e.g., “yes”) and the largest number within ordinal variables (e.g., for the Age variable: “aged 30–39,” n = 11,727, weighted 47.2 %) or nominal variables (e.g., for the Region variable: “South,” n = 6,914, weighted 39.9 %) as the reference group.

**Table 1:** Survey-weighted percentages of at least one dose of COVID-19 vaccine and likelihood of vaccination for children under 5 by sociodemographic information of the households.

**Characteristics of respondents**	**Unweighted No.** **(weighted %)**	**Weighted percentage (95 % CI)**						
		**Children vaccinated**	**Children unvaccinated: likelihood of getting vaccinated**					
		**At least 1 dose**	**Definitely**	**Probably**	**Unsure**	**Probably NOT**	**Definitely NOT**	**Don’t know**
Overall	21,698	24.5 (22.2–26.8)	6.0 (5.3–6.6)	6.3 (5.6–7.0)	10.0 (8.9–11.1)	12.7 (10.9–14.5)	29.8 (25.6–34.1)	10.6 (7.0–14.2)
**COVID-19 vaccination status (n = 21,678)**								
Yes (ref.)	17,310 (74.4)	31.6 (30.0–33.2)	7.7 (6.7–8.8)	8.1 (7.1–9.1)	12.0 (10.8–13.2)	13.1 (11.5–14.7)	17.4 (14.4–20.4)	10.1 (6.3–14.0)
No	4,368 (25.6)	3.9 (2.0–5.9)***	0.8 (0.3–1.4)***	1.2 (0.7–1.7)***	4.3 (3.1–5.5)***	11.6 (8.7–14.5)	66.0 (62.4–69.5)***	12.1 (14.0–15.6)

**COVID-19 status: Have ever tested positive for COVID-19 (n = 21,361)**								
Yes (ref.)	14,312 (62.9)	24.2 (22.9–25.4)	5.8 (5.0–6.5)	6.4 (5.6–7.3)	10.6 (9.1–12.2)	12.8 (9.7–15.9)	29.7 (26.6–32.9)	10.5 (6.4–14.6)
No	7,049 (37.1)	26.1 (21.8–30.4)	6.6 (5.4–7.7)	6.1 (4.9–7.4)	8.5 (7.2–9.9)**	12.4 (11.0–13.7)	29.1 (22.6–35.6)	11.2 (8.3–14.2)

**Presence of current COVID-19 symptoms (n = 13,544)**								
Yes (ref.)	1,363 (11.0)	24.9 (20.6–29.2)	5.9 (2.2–9.5)	7.8 (5.0–10.7)	10.8 (4.4–17.2)	12.0 (9.1–15.0)	27.2 (22.7–31.7)	11.4 (4.5–18.3)
No	12,181 (89.0)	24.7 (23.3–26.1)	5.8 (5.2–6.4)	6.3 (5.4–7.3)	10.8 (8.5–13.1)	12.9 (9.1–16.7)	29.4 (26.4–32.5)	10.1 (6.9–13.3)

**Race/ethnicity (n = 21,698)**								
Non-Hispanic White (ref.)	14,823 (52.2)	21.8 (20.3–23.2)	5.4 (4.6–6.2)	5.6 (4.8–6.4)	8.2 (7.2–9.2)	13.0 (12.0–13.9)	36.5 (34.3–38.7)	9.6 (5.9–13.2)
Non-Hispanic Black	1,815 (13.2)	22.4 (18.5–26.3)	5.0 (3.7–6.3)	7.0 (5.0–9.0)	10.7 (8.5–12.8)*	15.7 (12.7–18.7)	24.1 (17.5–30.6)***	15.1 (9.1–21.1)***
Non-Hispanic Asian	1,262 (6.0)	42.1 (36.4–47.8)***	10.6 (8.1–13.1)***	10.6 (7.8–13.5)***	10.7 (8.0–13.4)	10.2 (7.0–13.5)	7.7 (5.9–9.6)***	8.0 (5.3–10.6)
Non-Hispanic other race	1,126 (5.3)	24.6 (20.3–28.9)	5.0 (2.6–7.4)	3.7 (1.4–6.0)	8.9 (6.5–11.3)	9.9 (6.7–13.1)	33.2 (26.4–39.9)	14.8 (5.7–23.8)
Hispanic	2,672 (23.3)	27.3 (18.4–36.2)	6.8 (4.9–8.6)	7.1 (4.6–9.6)	13.8 (10.3–17.3)**	11.6 (4.8–18.5)	23.1 (18.9–27.4)***	10.3 (8.0–12.6)

**Sex at birth (n = 21,698)**								
Male (ref.)	8,520 (44.4)	28.5 (26.8–30.3)	6.1 (5.1–7.0)	6.5 (5.3–7.7)	7.5 (6.3–8.6)	11.4 (9.7–13.2)	30.8 (27.4–34.1)	9.2 (5.6–12.9)
Female	13,178 (55.7)	21.3 (18.4–24.2)***	5.9 (5.0–6.7)	6.2 (4.9–7.5)	12.0 (9.7–14.4)**	13.7 (11.6–15.8)*	29.1 (23.7–34.5)	11.8 (7.9–15.6)**

**Age (n = 21,698)**								
18–29	2,646 (20.1)	16.0 (6.2–25.9)	4.7 (3.1–6.2)*	6.0 (3.8–8.3)	10.8 (8.0–13.7)	15.2 (13.4–17.1)	35.3 (21.9–48.7)	11.9 (9.7–14.1)***
30–39 (ref.)	11,727 (47.2)	24.6 (22.8–26.5)	6.3 (5.6–7.1)	6.5 (5.7–7.3)	9.8 (8.8–10.8)	13.4 (11.0–15.7)	30.9 (29.4–32.3)	8.5 (7.1–9.8)
40–49	5,215 (19.2)	31.3 (25.0–37.6)	6.8 (5.5–8.2)	6.9 (4.7–9.1)	8.8 (7.6–10.1)	9.9 (8.5–11.3)*	27.2 (24.8–29.6)*	9.0 (3.8–14.3)
50–59	1,124 (6.5)	24.4 (13.7–35.2)	4.9 (1.6–8.1)	5.6 (3.1–8.0)	14.2 (9.1–19.4)	11.7 (8.1–15.4)	22.1 (18.4–25.9)***	17.0 (10.3–23.7)**
60–69	726 (5.1)	29.6 (20.6–38.7)	4.9 (0.2–9.6)	5.9 (3.0–8.8)	9.0 (4.1–13.9)	8.8 (6.2–11.4)*	19.4 (15.5–23.2)***	22.4 (17.8–27.0)***
70+	260 (1.8)	29.2 (19.3–39.0)	7.7 (2.3–13.1)	3.7 (0–18.2)	5.5 (1.4–9.5)	11.9 (2.9–20.9)	28.1 (17.4–38.8)	13.9 (1.2–26.7)

**Region (n = 21,698)**								
Northeast	3,118 (15.9)	26.4 (23.5–29.3)**	7.0 (5.1–9.0)	6.0 (4.4–7.6)	11.0 (8.6–13.5)	12.9 (10.6–15.2)	27.2 (23.8–30.5)***	9.5 (5.2–13.7)
South (reference)	6,914 (39.9)	19.9 (17.6–22.2)	6.2 (5.4–6.9)	6.9 (5.6–8.2)	9.5 (7.5–11.5)	13.0 (11.2–14.9)	33.7 (30.8–36.6)	10.8 (8.8–12. 9)
Midwest	4,896 (20.0)	25.0 (22.6–27.5)***	5.3 (4.4–6.2)	5.3 (4.1–6.5)	8.2 (7.0–9.3)	13.6 (12.3–14.8)	30.8 (22.8–38.9)	11.8 (5.6–18.0)
West	6,770 (24.2)	30.5 (26.4–34.6)***	5.5 (3.8–7.2)	6.5 (4.3–8.7)	11.7 (7.3–16.1)	11.3 (7.2–15.5)	24.4 (19.1–29.6)***	10.1 (5.5–14.7)

**Marital status (n = 21,662)**								
Now married (ref.)	16,957 (70.0)	27.2 (26.3–28.2)	6.2 (5.6–6.9)	6.3 (5.5–7.0)	9.7 (8.9–10.5)	12.0 (10.4–13.5)	29.8 (27.8–31.7)	8.8 (5.5–12.0)
Widowed	286 (1.9)	31.3 (19.4–43.3)	4.4 (0–9.2)	9.5 (2.3–16.7)	4.6 (2.0–7.3)***	9.1 (3.7–14.4)	20.1 (9.8–30.4)*	21.0 (12.9–29.1)**
Divorced	1,273 (5.7)	17.6 (10.8–24.4)**	8.3 (4.3–12.3)	4.5 (1.3–7.6)	11.6 (7.9–15.2)	11.1 (8.1–14.1)	32.2 (27.4–37.0)	14.8 (5.9–23.6)
Separated	387 (2.5)	19.2 (13.2–25.1)**	3.0 (0–6.0)*	8.7 (3.0–14.4)	19.9 (3.4–36.3)	13.9 (8.2–19.5)	24.1 (13.8–34.5)	11.3 (7.3–15.3)
Never married	2,759 (20.0)	16.8 (7.6–26.0)*	4.9 (3.8–5.9)*	6.5 (5.1–7.9)	10.0 (7.9–12.1)	15.8 (11.6–20.0)*	31.1 (18.5–43.6)	14.9 (10.3–19.6)***

**Educational attainment (n = 21,698)**								
Less than or some high school	566 (9.4)	28.5 (21.3–35.7)	5.9 (3.5–8.3)	8.1 (1.5–14.7)	14.2 (7.9–20.5)	11.8 (7.3–16.2)	19.5 (12.5–26.6)	11.9 (7.8–16.1)*
High school graduate or equivalent	2,512 (28.3)	17.5 (10.6–24.3)***	4.4 (3.2–5.7)***	4.2 (2.6–5.8)***	8.0 (6.8–9.2)*	13.9 (10.3–17.4)	38.2 (30.2–46.1)***	13.9 (8.1–19.6)**
Some college but degree not received or is in progress	3,998 (19.2)	16.2 (13.1–19.3)***	6.1 (4.8–7.4)	6.3 (4.1–8.4)	10.9 (8.8–13.1)	12.1 (9.2–14.9)	36.1 (30.3–41.9)***	12.4 (9.5–15.2)***
Associate’s degree	2,072 (9.6)	17.8 (12.8–22.9)***	6.1 (4.4–7.8)	5.9 (4.5–7.3)*	11.7 (9.3–14.1)	14.0 (14.5–16.6)	34.5 (28.1–40.8)***	10.0 (7.7–12.3)**
Bachelor’s degree (ref.)	6,613 (18.4)	30.3 (28.9–31.8)	6.9 (5.8–7.9)	7.8 (6.9–8.7)	9.8 (8.4–11.2)	13.2 (11.9–14.4)	24.5 (21.5–27.6)	7.5 (5.9–9.1)
Graduate degree	5,937 (15.1)	42.8 (40.2–45.4)***	7.5 (6.5–8.4)	7.9 (6.6–9.2)	9.2 (7.7–10.6)	10.5 (8.6–12.5)*	16.2 (14.7–17.7)***	5.9 (2.8–8.9)

**Household income (n = 17,142)**								
Less than $25,000	1,131 (10.5)	17.1 (13.5–20.6)**	8.5 (6.2–10.7)	5.1 (1.4–8.7)	13.4 (8.3–18.5)	11.6 (3.4–19.9)	28.2 (18.7–37.8)	16.1 (3.8–28.4)
$25,000–$34,999	990 (9.5)	20.8 (14.5–27.1)	5.1 (3.0–7.2)	5.0 (0–10.1)	10.5 (6.8–14.2)	12.0 (7.5–16.5)	31.6 (27.0–36.2)	15.0 (12.1–18.0)***
$35,000–$49,999	1,384 (11.8)	19.3 (11.3–27.3)	6.0 (3.9–8.1)	7.8 (4.2–11.3)	8.8 (5.3–12.2)	14.4 (10.6–18.3)	31.6 (18.7–44.5)	12.1 (9.2–15.1)*
$50,000–$74,999	2,341 (16.4)	19.4 (14.1–24.8)	6.2 (4.8–7.7)	6.0 (4.1–8.0)	12.9 (9.0–16.9)	14.9 (12.7–17.2)	31.3 (19.0–43.5)	9.2 (6.5–11.8)
$75,000–$99,999	2,416 (14.3)	20.4 (14.6–26.2)	5.2 (2.5–7.8)	6.1 (4.3–8.0)	10.1 (8.3–12.0)	12.0 (8.7–15.3)	34.9 (28.7–41.0)	11.3 (6.1–16.6)*
$100,000–$149,999 (ref.)	3,656 (17.0)	25.1 (21.9–28.2)	6.7 (4.9–8.4)	7.5 (6.2–8.7)	8.5 (7.0–10.0)	14.8 (12.9–16.6)	29.4 (27.0–31.7)	8.3 (4.6–11.9)
$150,000–$199,999	2,043 (8.9)	37.7 (34.2–41.3)***	6.2 (4.5–8.0)	7.9 (5.8–10.0)	8.6 (6.6–10.6)	10.0 (6.6–13.4)**	21.8 (17.8–25.7)***	7.8 (1.5–14.1)
$200,000 and above	3,181 (11.6)	50.3 (43.5–57.0)***	6.9 (4.3–9.5)	6.8 (4.9–8.6)	7.1 (5.7–8.6)	8.7 (7.1–10.2)***	16.0 (11.2–20.8)***	4.2 (1.2–7.2)***

**Covered by health insurance (n = 21,698)**								
Yes (ref.)	17,268 (71.8)	25.7 (23.6–27.9)	6.4 (5.5–7.3)	6.7 (5.9–7.5)	10.0 (8.6–11.3)	13.0 (11.6–14.5)	28.3 (23.8–32.7)	9.9 (6.6–13.2)
No	4,430 (28.3)	21.4 (17.5–25.3)**	4.8 (3.9–5.7)*	5.4 (4.1–6.7)	10.2 (8.6–11.8)	11.9 (8.9–14.9)	33.9 (29.0–38.8)***	12.5 (8.2–16.8)**

Statistically significant difference compared to the reference group from the test for difference in proportions at *P < 0.05, **P < 0.01, and ***P < 0.001.

Ref. means reference group in each variable.

Overall, 24.5 % (95 % CI: 22.2–26.8) of children under 5 received at least one dose of the COVID-19 vaccine (Table 1). This included 13.4 % (95 % CI: 12.3–14.6) who received the vaccine on or after December 9, 2022; 7.4 % (95 % CI: 6.0–8.8) who received it less than a year ago but before December 9, 2022; and 3.7 % (95 % CI: 1.9–5.5) who received it more than a year ago (Table 1 and Fig. S1 in Supplementary Material). Among households that had not vaccinated their children, 12.3 % expressed willingness to vaccinate (definitely: 6 %, 95 % CI: 5.3–6.6 and probably: 6.3 %, 95 % CI: 5.6–7.0), while 22.7 % displayed moderate hesitancy (unsure: 10 %, 95 % CI: 8.9–11.1 and probably NOT:12.7 %, 95 % CI: 10.9–14.5), and 29.8 % (95 % CI: 25.6–34.1) showed strong hesitancy. Respondents who had not received the COVID-19 vaccine were significantly less likely to have their children vaccinated with at least one dose of the vaccine (3.9 %, 95 % CI: 2.0–5.9, *P* < 0.001) and expressed significantly lower intention to vaccinate their children (definitely: 0.8 %, 95 % CI: 0.3–1.4, *P* < 0.001; definitely NOT: 66 %, 95 % CI: 62.4–69.5, *P* < 0.001) compared to respondents who had received the COVID-19 vaccine. In general, COVID-19 status (having ever tested positive for COVID-19) and the presence of current COVID-19 symptoms did not affect the likelihood of getting the children vaccinated.

We examined the likelihood of vaccinating or administering boosters to children under 5, categorized by race/ethnicity (Table 1 and Fig. S2 in Supplementary Material). To determine race/ethnicity, we utilized two questions from the HPS data: 1) “Are you of Hispanic, Latino, or Spanish origin?” and 2) “What is your race?” Based on these questions, we classified race/ethnicity into five categories: 1) non-Hispanic White, 2) non-Hispanic Black, 3) non-Hispanic Asian, 4) non-Hispanic other race, and 5) Hispanic. Among the five race/ethnicity groups, non-Hispanic Asian respondents showed the highest proportion of children under 5 in their households who received at least one dose of the COVID-19 vaccine across all three vaccinated periods (on or after December 9, 2022: 24.8 %, 95 % CI: 19.2–30.3, before December 9, 2022 but less than a year ago: 11.3 %, 95 % CI: 5.1–17.5, and more than a year ago: 6.0 %, 95 % CI: 0–12.2). Furthermore, non-Hispanic Asian respondents were significantly more likely to vaccinate their children and had the lowest proportion of strong hesitancy (7.7 %, *P* < 0.001). Conversely, non-Hispanic White respondents had the lowest proportion of children vaccinated with at least one dose of the COVID-19 vaccine and showed the highest proportion of strong hesitancy (36.5 %).

The weighted percentage of children under 5 who had received at least one dose of the COVID-19 vaccine is depicted based on household sex, region, marital status, educational attainment, income, and health insurance coverage (Table 1 and Fig. S3 in Supplementary Material). Female respondents had a significantly lower percentage of children under 5 with at least one dose of vaccine (21.3 %, 95 % CI: 18.4–24.2, *P* < 0.001) compared to male respondents. Furthermore, female respondents showed a significantly higher percentage of moderate hesitancy in vaccinating children (unsure: 12.0 %, 95 % CI: 9.7–14.4, *P* < 0.01; probably NOT: 13.7 %, 95 % CI: 11.6–15.8, *P* < 0.05) and a statistically significant higher percentage of “don’t know” responses (11.8 %, 95 % CI: 7.9–15.6) compared to male respondents. Respondents from the Northeast (26.4 %), Midwest (25.0 %), and West (30.5 %) regions were significantly more likely to have children under 5 with at least one dose of vaccine. In addition, those who had divorced (17.6 %, *P* > 0.01), separated (19.2 %, *P* < 0.01), and never been married (16.8 %, *P* < 0.05) displayed significantly lower percentages of children under 5 with at least one dose of the vaccine than respondents who are currently married. For educational attainment, as the level of education increased, the percentage of children under 5 with at least one dose of the vaccine generally increased. Likewise, respondents with an education level below a bachelor’s degree (excluding “Less than or some high school”) expressed significantly more strong hesitancy toward their children receiving vaccines, while respondents with an education level above a bachelor’s degree were less likely to express strong vaccine hesitancy.

There was an overall positive relationship between household income and the percentage of children with at least one dose of the vaccine: 17.1 % (95 % CI: 13.5–20.6) in the lowest income group (less than $25,000) compared to 50.3 % (95 % CI: 43.5–57.0) in the highest income group ($200,000 and above). As presented in Table 1, the two highest income groups had significantly lower percentages of strong vaccine hesitancy. Respondents without health insurance had significantly lower percentages of children under 5 with at least one dose of the vaccine (21.4 %, 95 % CI: 17.5–25.3, *P* < 0.001) compared to respondents with health insurance. Those without health insurance had a significantly higher percentage of strong vaccine hesitancy (definitely NOT: 33.9 %, 95 % CI: 29.0–38.8, *P* < 0.001) and no plans for vaccination (don’t know: 12.5 %, 95 % CI: 8.2–16.8, *P* < 0.01) and were less likely to express willingness to vaccinate their children (definitely: 4.8 %, 95 % CI: 3.9–5.7, *P* < 0.05). There were no significant differences in the percentage of children under 5 with at least one dose of vaccine based on the age of the respondents (Table 1). However, as the age group goes up between 40–49 to 60–69, the percentage of strong hesitancy (40–49 years: 27.2 %, 95 % CI: 24.8–29.6, *P* < 0.05; 50–59 years: 22.1 %, 95 % CI: 18.4–25.9, *P* < 0.001; and 60–69 years: 19.4 %, 95 % CI: 15.5–23.2, *P* < 0.001) significantly decreased, while the percentage of “don’t know” responses significantly increased (50–59 years: 17.0 %, 95 % CI: 10.3–23.7, *P* < 0.01; and 60–69 years: 22.4 %, 95 % CI: 17.8–27.0, *P* < 0.001).

### Reasons influencing vaccine hesitancy for children under 5 in households

We analyzed the reasons why households hesitate to vaccinate children under 5. The analysis was based on a sample size of 11,787 respondents whose children under 5 had not received the COVID-19 vaccine or booster. They provided responses indicating their likelihood of getting their children vaccinated as “probably,” “unsure,” “probably NOT,” or “definitely NOT.” The results indicated that more than half of the respondents (52.4 %, 95 % CI: 48.0–56.8) expressed concerns about potential side effects from the vaccine. Approximately one-third of respondents (32.9 %, 95 % CI: 30.7–35.3) reported a lack of trust in the COVID-19 vaccines. Other commonly cited reasons included perceiving children as not being in high-risk groups (29.3 %, 95 % CI: 24.8–33.7), not believing that children need a vaccine (28.4 %, 95 % CI: 25.1–31.6), and intending to wait and observe the safety of the vaccine (27.1 %, 95 % CI: 24.8–33.7). Additionally, reasons such as distrust in the government (25.0 %, 95 % CI: 22.8–27.1), and the absence of a recommendation from the children’s doctor (17.6 %, 95 % CI: 16.6–18.5) were mentioned as reasons contributing to the decision not to vaccinate children.

### Likelihood of vaccinating children under 5 based on reasons of vaccine hesitancy

**Fig. 2:**
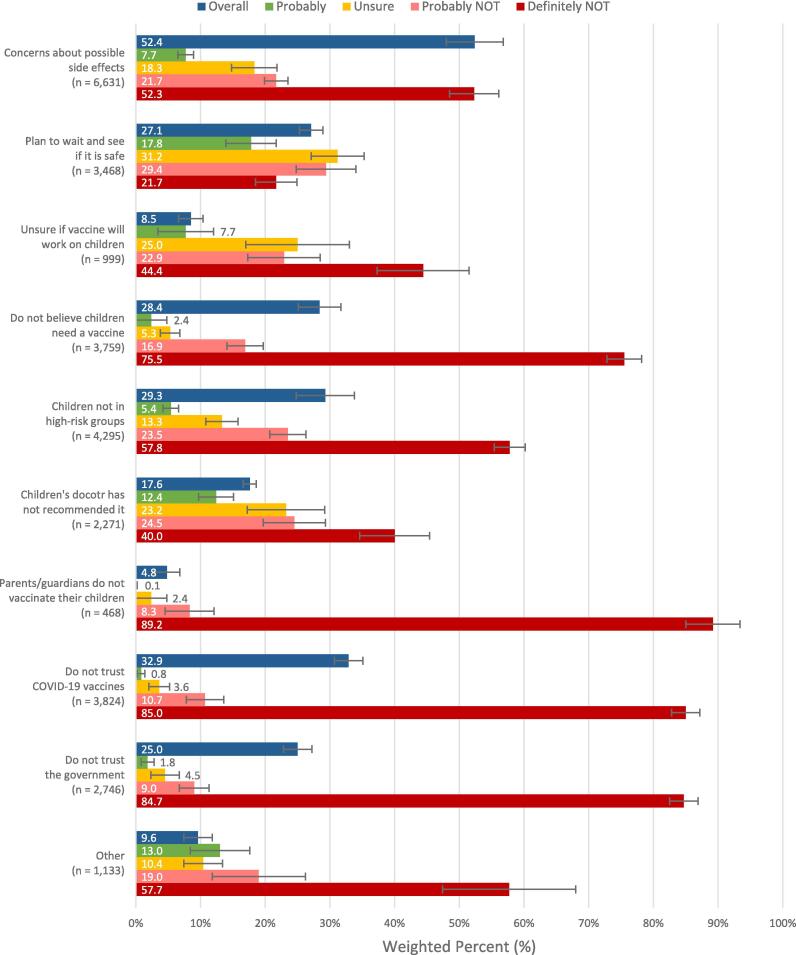
Households’ intention to vaccinate children under 5 based on why not vaccinating children under 5 (n = 11,787).

We conducted an analysis of the distribution of the likelihood of vaccinating children under 5 based on reasons for vaccine hesitancy (Fig. 2). When comparing strong hesitancy to moderate hesitancy concerning the vaccination of children, significant differences were observed for almost all of the reasons (9 out of 10), except for the reason “plan to wait and see if it is safe.” Respondents who chose “definitely NOT” likely made a firm decision against vaccination and were less inclined to wait and observe vaccine safety, which is reflected in the lowest percentage for the reason “plan to wait and see if it is safe” (21.7 %, 95 % CI: 18.5–24.9). The top three reasons for strong hesitancy in vaccinating children were “parents/guardians do not vaccinate their children” (89.2 %, 95 % CI: 85.0–93.4), “lack of trust in COVID-19 vaccines” (85.0 %, 95 % CI: 82.8–87.1), and “lack of trust in the government” (84.7 %, 95 % CI: 82.5–86.9). These reasons primarily stemmed from parents or guardians deciding not to vaccinate their children due to a lack of trust in COVID-19 vaccines and the government.

### Reasons for vaccine hesitancy regarding updated COVID-19 booster dose among household respondents

HPS includes data on the reasons for not receiving an updated COVID-19 booster dose among household respondents. To determine whether households received the booster, the survey asked the question, “how long ago was your recent dose of the COVID-19 vaccine or booster?” to those who received the COVID-19 vaccine (n = 11,068). The question provided three multiple-choice options: 1) on or after September 1, 2022, 2) before September 1, 2022 but less than a year ago, 3) more than a year ago. The data considered households that selected the second and third options as not having received the vaccine booster yet, which corresponds to the updated (bivalent) booster vaccine available from September 2, 2022. Regarding the reasons why households would not get the booster vaccine, participants were able to select more than one answer. Among them, almost one-third of respondents had not received an updated COVID-19 booster dose because they already had COVID-19 (31.2 %, 95 % CI: 28.7–33.7). Similarly, more than a quarter of respondents believed they already had sufficient immunity against COVID-19 from their original dose of the vaccine and have not received a booster dose, even if they were eligible and planned to get it, 26.3 % (95 % CI: 23.9–28.6) and 26.0 % (95 % CI: 23.4–28.5), respectively. Additionally, respondents indicated that they did not feel obligated to get the booster vaccine because they were not worried about getting COVID-19 (23.9 %, 95 % CI: 19.4–28.4) or because it was not required by their work or school (18.6 %, 95 % CI: 16.3–20.8).

## Discussion

This study provided insights into COVID-19 vaccine or booster intake and hesitancy for children under 5, by analyzing the HPS Phase 3.8 data conducted by the U.S. Census Bureau. We categorized the likelihood of vaccination into three definitions: willingness to vaccinate (definitely or probably), moderate hesitancy (unsure or probably NOT), and strong hesitancy (definitely NOT), providing clear and mutually exclusive levels of vaccine hesitancy, while CDC used different three categories: strongly hesitant (definitely not), hesitant (probably not or definitely not), and hesitant or unsure (probably not, unsure, or definitely not) [33]. Children under 5, especially those with frail conditions, are at an increased risk of COVID-19 infections. Contrary to the CDC’s recommendation for vaccination, our findings indicated significantly higher COVID-19 vaccine hesitancy for children under 5 in the United States. Among all participants, more than 50 % expressed hesitancy regarding vaccinating children under 5 (52.5 % overall: 29.8 % with strong hesitancy and 22.7 % with moderate hesitancy) (Fig. S1 in Supplementary Material). In comparison to the previous coverage of 10.1 % with at least one COVID-19 vaccine dose among children under 5 as of December 31, 2022 [34], however, our findings showed a notable increase in coverage to 24.5 % (95 % CI: 22.2–26.8) in March–May 2023.

COVID-19 vaccine or booster uptake and hesitancy among households with children under 5 varied by sociodemographic factors. Unvaccinated respondents showed a statistically significant unwillingness to vaccinate children under 5, consistent with Sehgal et al.’s (2022) findings [20]. Similar to the findings in recent studies [35], [36], non-Hispanic Asian respondents had the highest rates of COVID-19 vaccine uptake and were least likely to show strong hesitancy towards vaccinating their children under 5 compared to respondents who were non-Hispanic White. Consistent with prior investigations focusing on children of older ages [35], [37], our findings similarly reflected reduced vaccination rates and heightened levels of strong hesitancy among individuals with lower educational attainment, lower household income, and no health insurance coverage. It is important to acknowledge, however, that the dynamics surrounding vaccine acceptability can be context-dependent and multifaceted. In contrast to our results, a study conducted in China reported a counterintuitive trend, indicating that higher education levels and greater income were inversely associated with parental COVID-19 acceptability for children aged 6–35 months [38]. These divergent outcomes emphasize the need for a nuanced understanding of sociocultural influences and regional variations, highlighting the intricate interplay between socioeconomic factors and vaccination attitudes. Globally, the COVID-19 vaccine hesitancy among adults in June 2022 was 20.9 %, showing a gradual decline from 28.5 % in June 2020 and 24.8 % in June 2021, but it remains substantial due to misinformation about vaccinations [39], [40]. A study conducted in Italy explored parents’ inclination to vaccinate their children with frail conditions, revealing a notably lower willingness of 29.9 % to vaccinate such vulnerable children against COVID-19 [41]. In Ireland, 50.6 % expressed the intention to vaccinate their child, 28.7 % stated they had no intention to vaccinate, and 20.2 % remained uncertain [42].

**Fig. 3:**
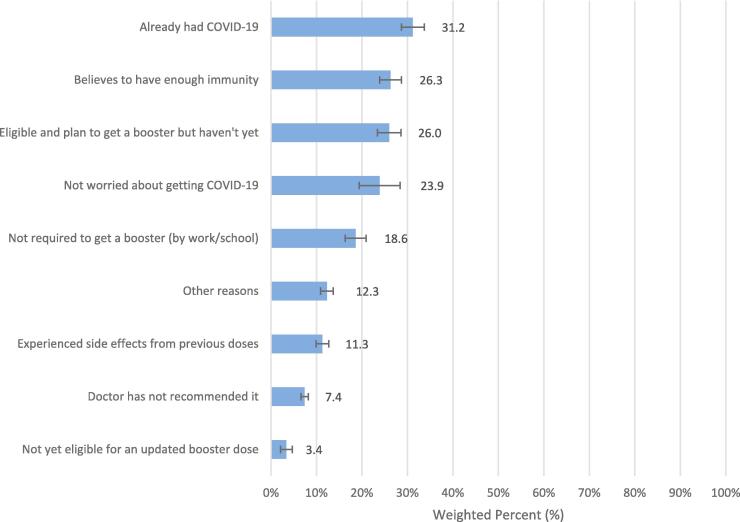
Reasons why household respondents have not received an updated COVID-19 booster dose (n = 11,068).

Our study identified common reasons for hesitancy in vaccinating of children, including concerns about safety, mistrust towards vaccines and the government, and perceptions of the vaccine being unnecessary for children, which align with previous research findings [35], [43], [44], [45]. Similarly, Bullock et al. (2022) reported reasons for booster refusal, attributing to perceptions that the second vaccine suffices for safety (59 %) or that the booster confers no additional protection (49 %), alongside apprehensions about potential long-term health impacts (33 %) and considerations about prioritizing the booster for others (22 %) [46]. In 2019, a conspiratorial mindset opposing COVID-19 vaccination was already prevalent in the United States, even before the pandemic emerged [47]. This disposition might have contributed to a scenario in which individuals with vaccine hesitancy had significantly less exposure to television and radio sources for pandemic-related information and displayed diminished trust in newspapers, television broadcasts, radio broadcasts, their medical practitioner, and other healthcare professionals [48]. Many respondents questioned the urgency or importance of vaccinating young children against COVID-19, possibly due to the perception that young children are at lower risk of severe illness (Fig. 2). Furthermore, previous research indicated that prior COVID-19 infection is a predictor of vaccine or booster uptake and hesitancy [49]. In our study, we found that 31.2 % (95 % CI: 28.7–33.7) of households reported not receiving an updated booster because they already had COVID-19 and believed they already obtained enough immunity against the virus (Fig. 3). Individuals may feel less compelled to get an updated booster dose due to the absence of booster vaccine mandates from authorities [50], [51], and our results showed the similar pattern.

There has been a noticeable absence of COVID-19 or booster vaccinations among children under 5, along with limited knowledge regarding vaccine hesitancy in this age group. Therefore, it is important to address the concerns contributing to COVID-19 vaccination status and vaccine hesitancy and to establish the robust safety track record of vaccines, empowering the public to make informed vaccination choices [52]. These collective insights underscore the intricate web of collaboration necessary to cultivate a well-informed and engaged society, actively participating in thoughtful vaccination decisions. In addition to the role of parents and guardians in ensuring children’s vaccination, healthcare providers can play pivotal roles as sources of information [53], [54]. As Carrieri et al. (2023) highlighted, trust emerges as an essential factor influencing vaccine hesitancy, suggesting the potential effectiveness of pro-vaccine campaigns directed specifically at high-risk hesitant groups [55]. Thus, it is evident to design comprehensive and well-coordinated health promotion campaigns, aiming to raise awareness about the significance of vaccines [52]. By focusing on building trust, addressing misconceptions, and providing clear and easily understandable information, public health efforts can reduce vaccine hesitancy and encourage informed vaccination decisions for children under 5.

### Limitations

The findings of this study are subject to a few limitations. First, we solely used the publicly available secondary data, HPS, which constrained our analysis to pre-existing information without the opportunity to customize data collection methods or address specific research questions through tailored data gathering. This reliance on existing datasets potentially restricts the depth of our findings and prevents us from exploring nuances that might have been emerged with more targeted or primary data collection approaches. For example, there was inconsistent information in the Phase 3.8 data (Weeks 55–57) that we used, specifically regarding the date entry, “most recent dose of the COVID-19 vaccine or booster children: On or after October 15, 2022 and Before October 15, 2022 but less than a year ago,” as stated in the Data Dictionary. This date discrepancy was not aligned with the booster vaccine availability date for children under 5, which was actually December 9, 2022. We found it to be an error based on the more recent data in Week 58. Consequently, we opted to use the date of December 9, 2022. This correction was imperative because updated vaccines for children under 5 were introduced, starting December 9, 2022, before the Phase 3.8 data were collected.

Second, the generalizability of these findings is limited due to low response rates across Weeks 55–57 of Phase 3.8 of HPS (6.7 %, 5.7 %, and 5.5 %, respectively), which may not fully represent the general population of children under 5 in the United States. However, this trend is evident in data from national surveys. For instance, a study in Ireland on parental attitudes toward childhood vaccination reported a response rate of 2.4 % among potential respondents, with only 10.1 % of them completing the national survey [42], demonstrating a notably lower response rate compared to that of the HPS data we utilized. To alleviate bias between the true value and sample estimates, the U.S. Census Bureau used successive difference replication to estimate standard errors in the HPS data [30]. In our study, we incorporated the 80 replicate weights from the HPS data. These techniques mainly assessed the magnitude of sampling errors, including some nonsampling effects, such as nonresponse and undercoverage. While they captured certain impacts of nonsampling error, they did not assess systematic biases associated with nonsampling error. Bias, in this context, represents the average discrepancy between sample estimates and the true value across all possible samples.

Third, individuals aged 18 and older from the household were eligible to participate in the study. An important consideration arises regarding the composition of diverse participants from the households; it is worth acknowledging that these individuals may not invariably serve as the genuine parents or guardians of children under 5. For example, if respondents were individuals who are not ultimately responsible for making vaccine decisions for the children in the household, their intentions and answers may be less relevant to the plans that parents have their children. Consequently, their level of awareness concerning the vaccination status of these young children may be incomplete, potentially introducing recall bias into the analysis. This bias could stem from their limited ability to accurately recollect or report vaccination information, impacting the overall accuracy and reliability of the findings.

Fourth, all the information provided by the respondents was self-reported, which introduced the possibility of social desirability bias. Lastly, HPS did not ask about vaccines and boosters separately. Instead, the question asked, “For the children in this household, how long ago was their most recent dose of the COVID-19 vaccine or booster?” This approach hindered the separate analysis of vaccine and booster uptake.

## Conclusions

The analysis of the HPS data highlights the presence of vaccine hesitancy among households with children under 5. Concerns about side effects, a lack of trust in vaccines and government, and the perception that children are not in high-risk groups were identified as key factors associated with hesitancy regarding the vaccination of children. Additionally, a significant proportion of households had not received the updated COVID-19 booster dose, with reasons ranging from ineligibility to perceived immunity from prior infection, which may affect their decision to vaccinate children under 5. These findings underscore the need for targeted interventions to address vaccine hesitancy, build trust, and promote the importance of booster doses, particularly focusing on specific concerns and beliefs of hesitant households with children under 5.

## CRediT authorship contribution statement

**Chulwoo Park:** Writing – review & editing, Writing – original draft, Visualization, Validation, Supervision, Software, Resources, Project administration, Methodology, Investigation, Formal analysis, Data curation, Conceptualization. **Pyramida Vagoyan Zabala:** Writing – original draft, Visualization, Formal analysis.

## Funding

None.

## Declaration of competing interest

The authors declare that they have no known competing financial interests or personal relationships that could have appeared to influence the work reported in this paper.

## Data Availability

Data will be made available on request.
