# Supplementary material for: COVID-19 vaccine or booster uptake and hesitancy for children aged 6 months–5 years in the United States: A national descriptive study using the household pulse survey between March and May 2023
Source: Vaccine X. 2024 Nov 7;21:100582. doi: 10.1016/j.jvacx.2024.100582 (PMC11609509; doi:10.1016/j.jvacx.2024.100582)

Figure S1. Weighted percentage of at least one dose of COVID-19 vaccine and intent for vaccination of children under 5, by household respondents' COVID-19 vaccination status, COVID-19 status, and presence of current COVID-19 symptoms

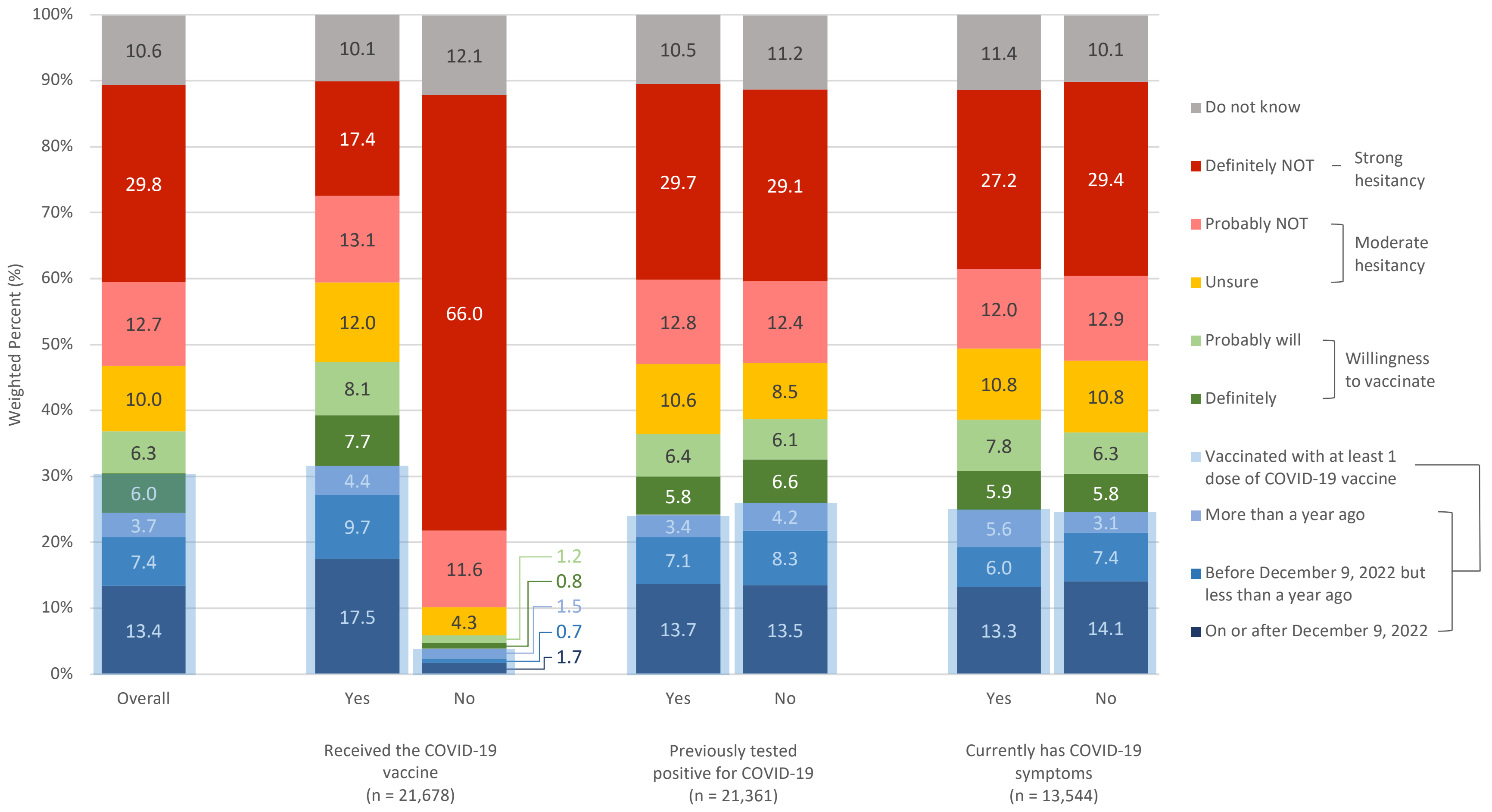

Figure S2. Weighted percentage of at least one dose of COVID-19 vaccine and intent for vaccination of children under 5, by household respondents' race/ethnicity

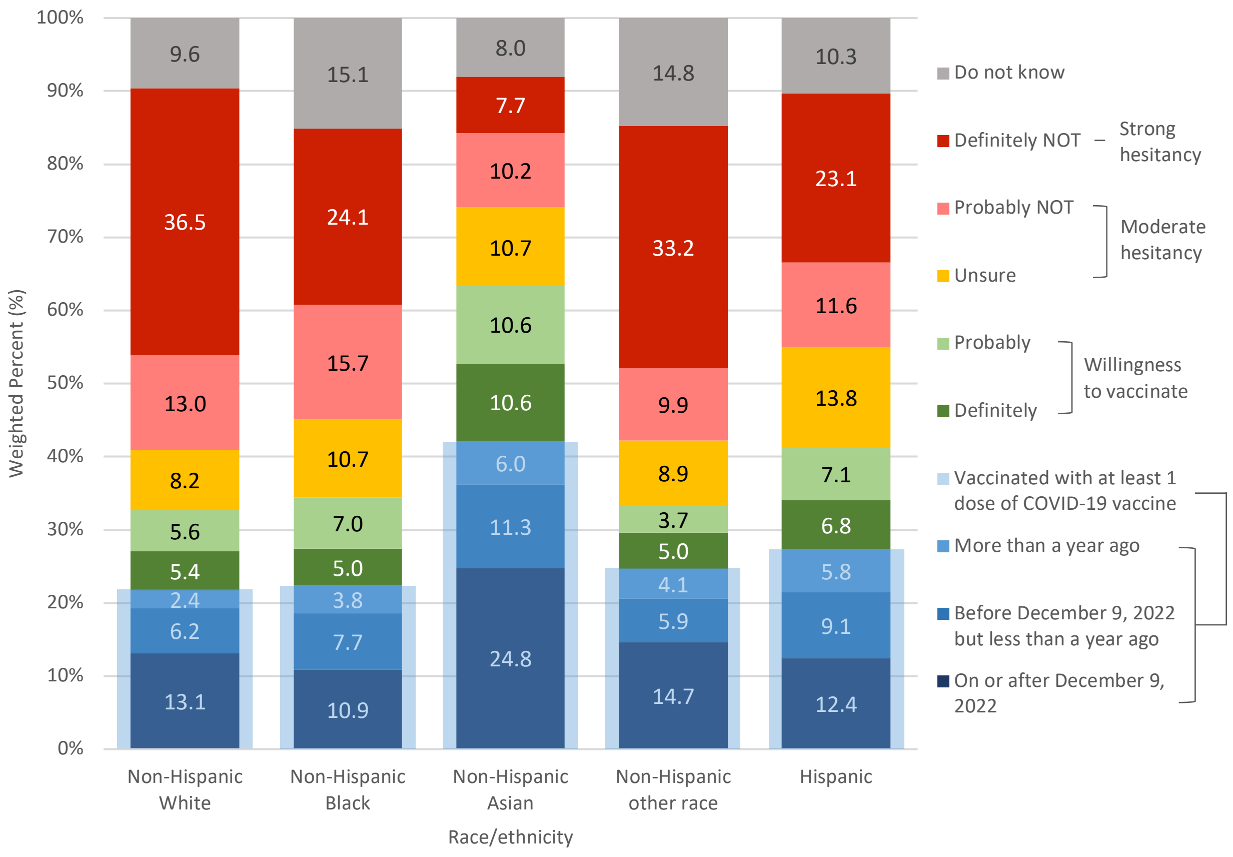

NOTE: N = 21,698

Figure S3. Weighted percentage of children under 5 vaccinated with at least one dose of COVID-19 vaccine, by household respondents' gender, region, marital status, educational attainment, income, and health insurance coverage

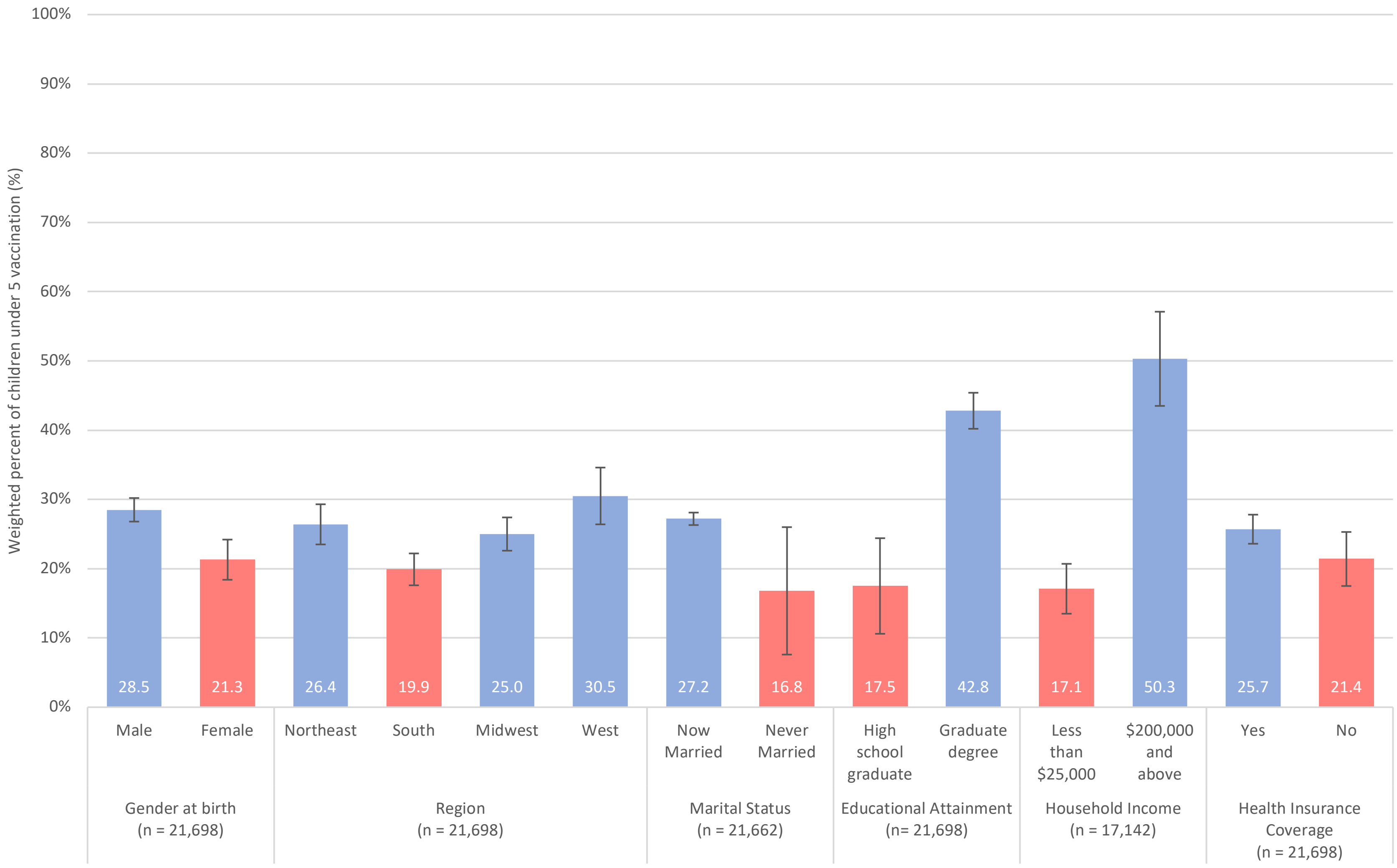

Supplement: Supplementary Data 1 [file mmc1.pdf]
